# Supplementary material for: Community diversity and habitat structure shape the repertoire of extracellular proteins in bacteria
Source: Nat Commun. 2020 Feb 6;11:758. doi: 10.1038/s41467-020-14572-x (PMC7005277; doi:10.1038/s41467-020-14572-x)
Supplement: Supplementary file 1 — Supplementary Information [file 41467_2020_14572_MOESM1_ESM.pdf]

Supplementary information for:

Community diversity and habitat structure shape the repertoire of extracellular proteins in Bacteria

Authors: Marc Garcia-Garcera[1,+], Eduardo PC Rocha[1]
[1] Microbial Evolutionary Genomics, Institut Pasteur, CNRS, UMR3525, 28, rue Dr Roux, Paris, 75015, France.
[+] present address: Department of Fundamental Microbiology. University of Lausanne. Batiment Biophore, Quartier SORGE, 1003, Lausanne.

THIS SUPPLEMENTARY FILE CONTAINS:

SUPPLEMENTARY FIGURES 2
SUPPLEMENTARY TABLES 7

Supplementary Figures

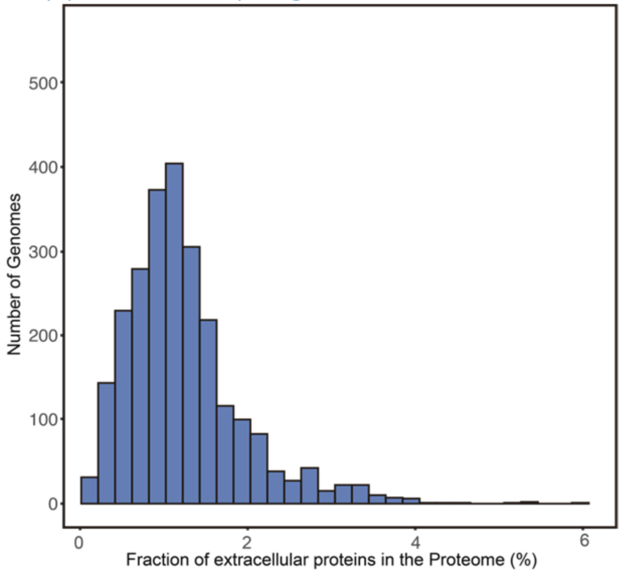

Supplementary figure 1. Histogram of the frequency of genes encoding extracellular proteins per genome.

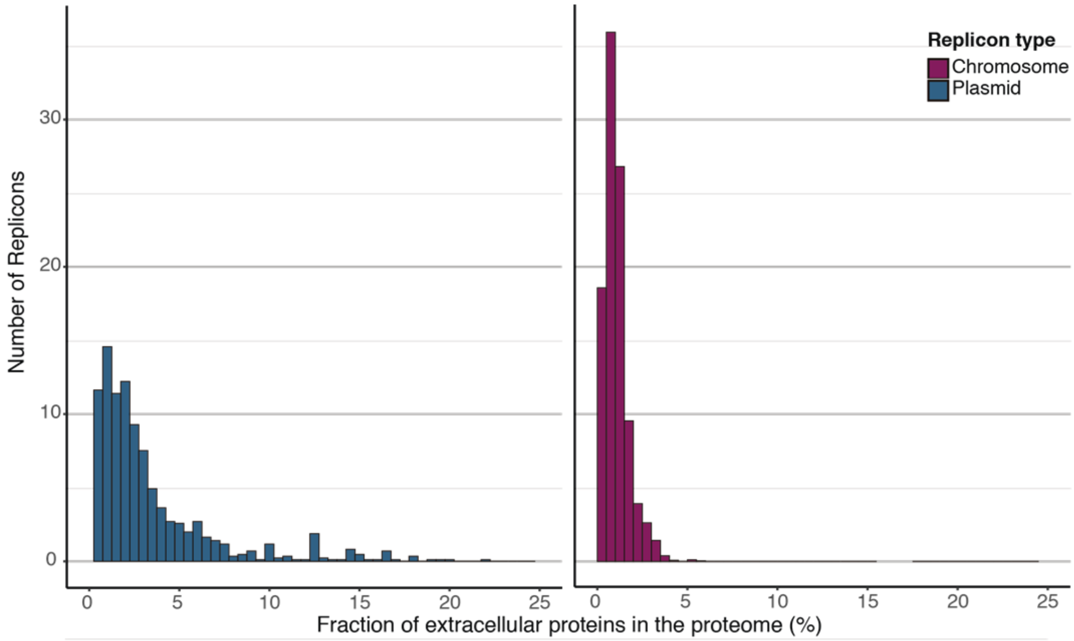

Supplementary figure 2. Frequency of genes encoding extracellular proteins in replicons. The replicons are coloured in function of their type. Values in the X axis were divided by the proteome size to remove its effect on the number of extracellular genes. The different colours represent the different types of replicons (Purple: Chromosomes; Blue: Plasmids).

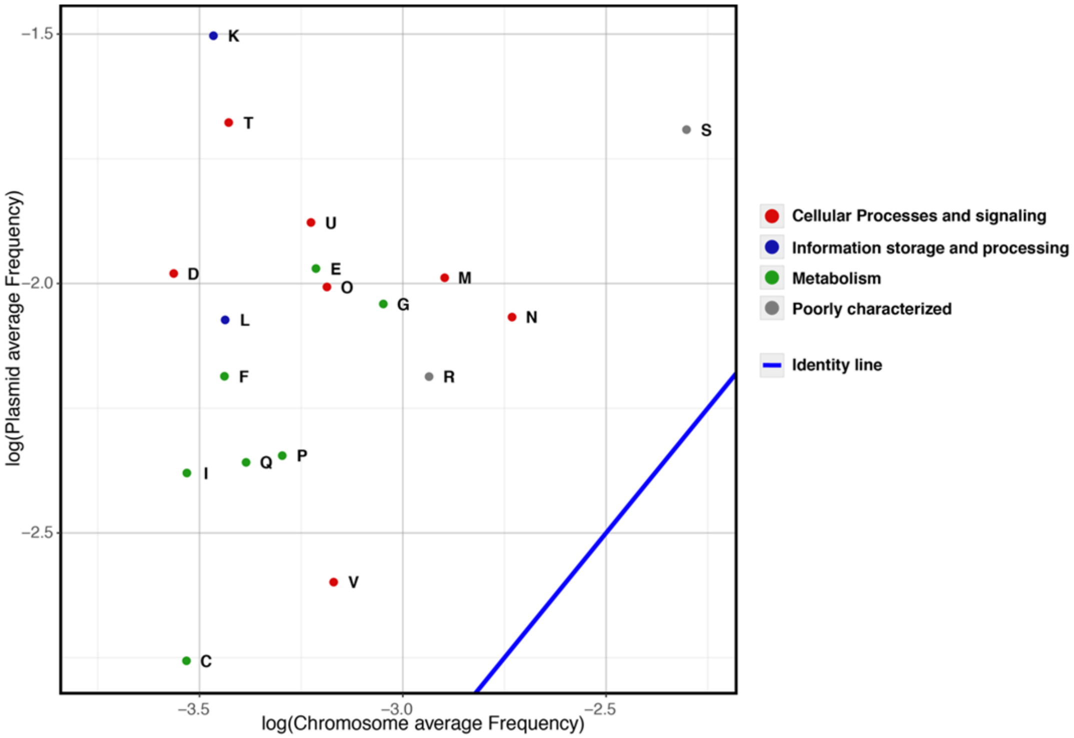

Supplementary figure 3. Frequency of extracellular proteins in chromosomes (X-axis) compared to the Frequency in plasmids (Y-axis). The blue line represents the identity line between both frequencies. Each dot represents the different COG functional categories (specified by the letter identifier), while colours represent the general class, associated to each function. P-values of the comparative analysis (Wilcoxon-rank test) are shown in **Supplementary table 5.**

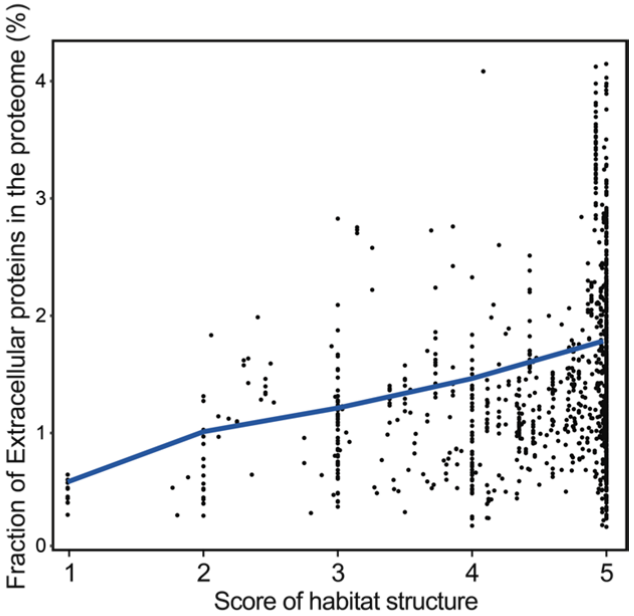

Supplementary figure 4. Average frequency of genes encoding extracellular proteins in genomes in function of the habitat structure score. Blue line

represents the mean Fraction of extracellular proteins in the proteome associated to each habitat bin in Figure 2a.

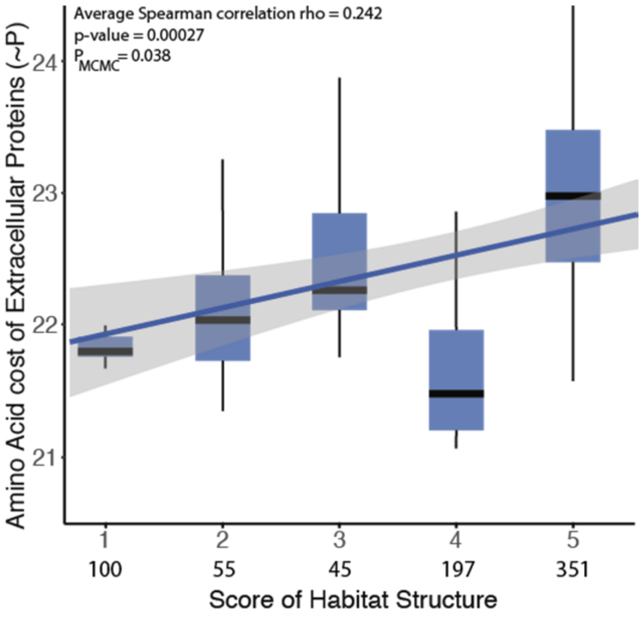

Supplementary figure 5. Association between the average amino acid cost of extracellular proteins and the score of habitat structure in Bacteria found in only one sub-category. Each boxplot represents the distribution of average amino acid costs for extracellular proteins in individual genomes. Numbers below the x-axis represent the number of genomes on each category (Spearman Correlation Rho = 0.24, P_{MCMC} < 0.05, regression statistics: a=0,12, b=21.67, r^2=0.098, P<0.05).

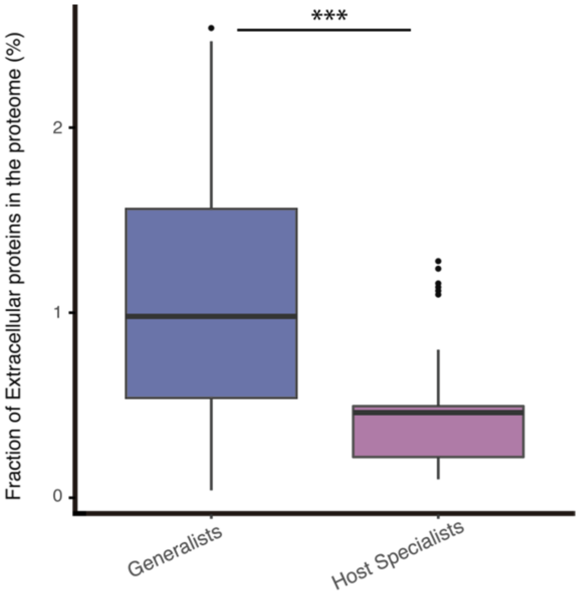

Supplementary figure 6. Association between extracellular proteins and lifestyle. Average frequency of extracellular proteins in generalist (dataset C, see supplementary materials and methods) and host-specialist (dataset B) species identified. Statistics reflect significant differences in the frequency of extracellular proteins and the different lifestyles. Wilcoxon-Rank test. ***, p-value < 0.0001.

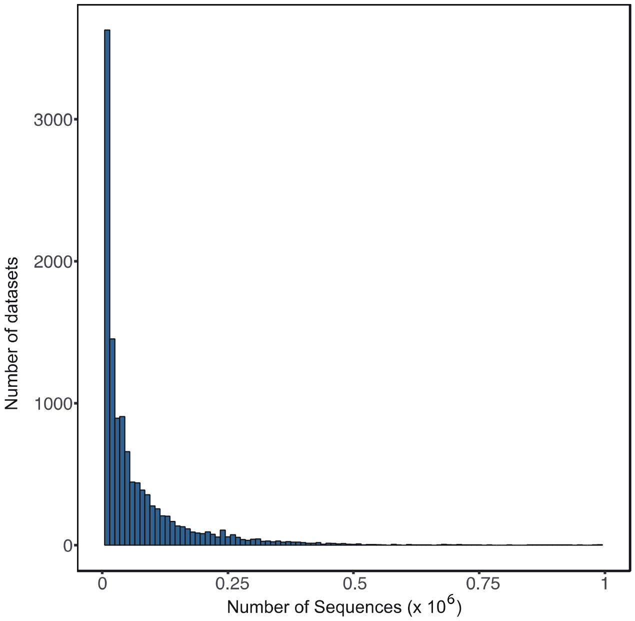

Supplementary figure 7. Distribution of the number of 16S rRNA sequences on each dataset.

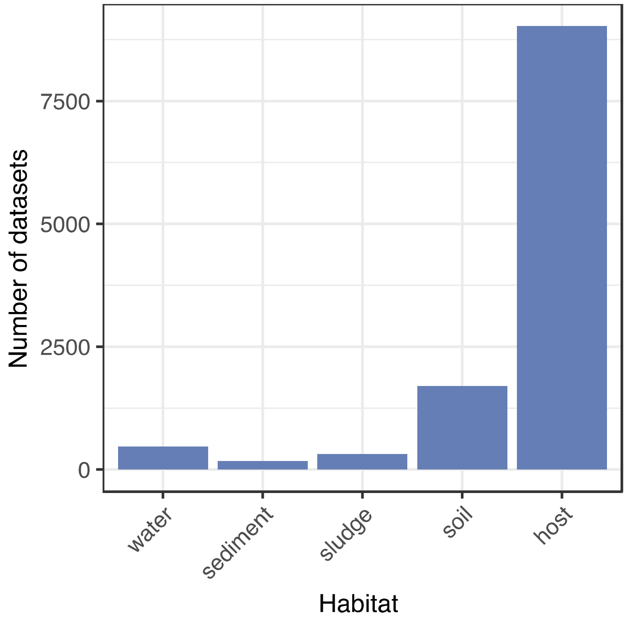

Supplementary figure 8. Number of datasets and their habitat classification.

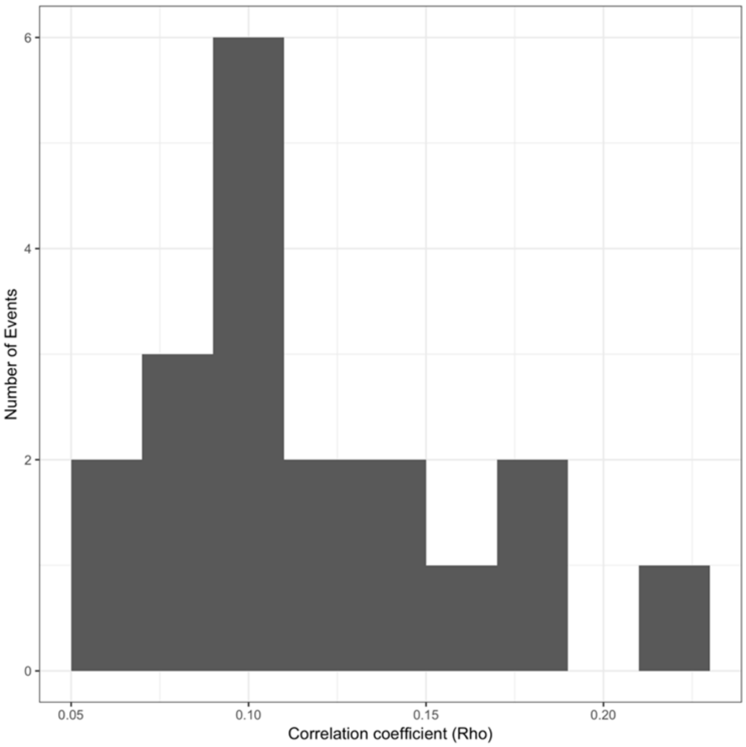

Supplementary figure 9. Histogram of the correlation coefficients between the diffusion length of extracellular proteins and the habitat structure score for each of the 25 different functional subcategories. All correlations were significantly different from zero (P<0.05), except for category D (cell cycle & mitosis), L (replication & repair) and H (coenzyme metabolism).

Supplementary tables

ENV1	ENV2	Freq	used in the study
**air**	indoor	11	NO
**air**	outdoor	42	NO
**biofilm**	hypersaline	3	NO
**biofilm**	microbial_mat	34	NO
**biofilm**	Hotsprings	130	NO
**biofilm**	inorganic surface	11	NO
**biofilm**	unclassified	76	NO
**bioreactor**	aerobic	72	NO
**bioreactor**	anaerobic	35	NO
**host**	animal	3657	YES
**host**	chorals	4	YES
**host**	arthropod	12	YES
**host**	human	3676	YES
**host**	mammal	192	YES
**host**	mixed	17	YES
**host**	other	15	YES
**host**	plant	931	YES
**host**	undefined	519	NO
**miscellaneous**	food	119	NO
**miscellaneous**	undefined	134	NO
**sediment**	freshwater	141	YES
**sediment**	marine	22	YES
**sediment**	spring	7	YES
**soil**	agricultural	9	YES
**soil**	desert	88	YES
**soil**	forest	661	YES
**soil**	grasslands	455	YES
**soil**	tundra	56	YES
**soil**	undefined	431	NO
**wastewater**	compost	32	YES
**wastewater**	treatment_plant	10	YES
**wastewater**	undefined	267	NO
**water**	freshwater	64	YES
**water**	house_associated	8	YES
**water**	marine	121	YES
**water**	undefined	5	NO

Supplementary Table 1. List of environmental classification categories and subcategories.

Amino Acid	Cost
**Ala**	11.7
**Cys**	24.7
**Asp**	12.7
**Glu**	15.3
**Phe**	52.0
**Gly**	11.7
**His**	38.3
**Ile**	32.3
**Lys**	30.3
**Leu**	27.3
**Met**	34.3
**Asn**	14.7
**Pro**	20.3
**Gln**	16.3
**Arg**	27.3
**Ser**	11.7
**Thr**	18.7
**Val**	23.3
**Trp**	74.3
**Tyr**	50.0

Supplementary table 2. Average amino-acid costs obtained from Akashi & Gojobori[26].

1. Bacteriocines

Identifier	Description
1	Linocin-like
2	Helveticin-like
3	Colicin E9
4	Colicin B
5	Colicin E1
6	Colicin Ia
7	Colicin K
8	Colicin U
9	Colicin A
10	Enterolysin A
11	Halocin-S8-like
12	Klebicin-like
13	Pesticin
14	Pyocin S1
15	Pyocin S2

16	Anacyclamide-like
17	Ancovenin
18	Nisin-like
19	BhtA1-like
20	BhtA2-like
21	Bottromycin
22	Bovicin
23	Cypermycin
24	Cytolysin-like
25	Glyocin-like
26	Plantaricin-W-like
27	Lantibiotic-A-like
28	Lariatin
29	Microcin-24-like
30	Microcin-VL-like
31	Patellamide-like
32	Siamycin
33	Linardin-like
34	Thiomuracin-Thiocillin
35	Thuricin
36	Pentocin-lik
37	Lcactocyclicin
38	Enterocin-like
39	Acidocin
40	Aureocin
41	BlpU-like
42	BlpK
43	BlpM
44	Colicin V
45	Curvaticin
46	Enterocin-L50-like
47	Lactococcin-G
48	Lichenin
49	Plantaricin-K
50	Plantaricin_NC8-like
51	Plantaricin-S
52	Putative-Bacteriocin-undefI
53	Thermophilin13-like

2. Degradative enzymes

Identifier	Description
AMD	Amidase (Prokaryotic)

AMYL	Amylase (Prokaryotic)
CELL	Cellulase (Prokaryotic)
INU	Inulinse (Prokaryotic)
INV	Invertase (Prokaryotic)
KER	Keratinase (Prokaryotic)
LIP	Lipase (Prokaryotic)
XYL	Xylanase (Prokaryotic)

Supplementary table 3. HMM profile identifiers for degradative enzymes and bacteriocins

question	test	value	p-value (adjusted)	associated figure
Differences between Frequency of Extracellular proteins in the different habitat categories	Wilcoxon Rank Test	NA	all < 0.001	Figure 2
Differences between Frequency of Extracellular proteins and score of habitat structure	Tukey HSD test	NA	all < 0.05	Figure 2
Differences between Frequency of Extracellular proteins and score of habitat structure	Spearman correlation	rho = 0.21	1,00E-17	Figure S3
Diffusion length vs Score of habitat structure	Spearman correlation	rho = 0.17	0.00058	Figure 3

Generalists vs Specialists (no host-associated)	Wilcoxon Rank Test	NA	0.0089	Figure 6
Amino Acid cost vs Score of habitat stucture	Spearman correlation	rho = 0.13	0.0018	Figure S4

Supplementary table 4. Statistical test results when removing host as environmental class

Functional Category	P-value (one-sided)	P-value FDR adjusted
**C**	1.666667e-01	1.875000e-01
**D**	5.000000e-01	5.000000e-01
**E**	4.329253e-06	7.084232e-06
**F**	1.726262e-09	3.884089e-09
**G**	3.575059e-07	7.150119e-07
**I**	1.000000e-01	1.200000e-01
**K**	5.000000e-01	5.000000e-01
**L**	2.088982e-14	6.266946e-14
**M**	6.929606e-43	6.236645e-42
**N**	3.139225e-06	5.650606e-06
**O**	8.717804e-19	3.138409e-18
**P**	2.921085e-04	4.381628e-04
**Q**	4.925729e-12	1.266616e-11
**R**	1.913863e-30	8.612383e-30
**S**	8.694394e-46	1.564991e-44
**T**	5.000000e-02	6.428571e-02
**U**	2.644254e-38	1.586552e-37
**V**	3.968254e-03	5.494505e-03

Supplementary table 5. Wilcoxon-rank test of the comparison between the frequency of extracellular proteins (by functional COG category) in chromosomes and plasmids.
