# Supplementary material for: Community diversity and habitat structure shape the repertoire of extracellular proteins in bacteria
Source: Nat Commun. 2020 Feb 6;11:758. doi: 10.1038/s41467-020-14572-x (PMC7005277; doi:10.1038/s41467-020-14572-x)
Supplement: Supplementary file 3 — Reporting Summary [file 41467_2020_14572_MOESM3_ESM.pdf]

nature research

Corresponding author(s): Garcia-Garcera, Marc

Last updated by author(s): 11.01.2020

Reporting Summary

Nature Research wishes to improve the reproducibility of the work that we publish. This form provides structure for consistency and transparency in reporting. For further information on Nature Research policies, see Authors & Referees and the Editorial Policy Checklist .

Statistics

For all statistical analyses, confirm that the following items are present in the figure legend, table legend, main text, or Methods section.

n/a	Confirmed	
☐	☒	The exact sample size (*n*) for each experimental group/condition, given as a discrete number and unit of measurement
☐	☒	A statement on whether measurements were taken from distinct samples or whether the same sample was measured repeatedly
☐	☒	The statistical test(s) used AND whether they are one- or two-sided<br>*Only common tests should be described solely by name; describe more complex techniques in the Methods section.*
☐	☒	A description of all covariates tested
☐	☒	A description of any assumptions or corrections, such as tests of normality and adjustment for multiple comparisons
☐	☒	A full description of the statistical parameters including central tendency (e.g. means) or other basic estimates (e.g. regression coefficient) AND variation (e.g. standard deviation) or associated estimates of uncertainty (e.g. confidence intervals)
☐	☒	For null hypothesis testing, the test statistic (e.g. *F*, *t*, *r*) with confidence intervals, effect sizes, degrees of freedom and *P* value noted<br>*Give P values as exact values whenever suitable.*
☒	☐	For Bayesian analysis, information on the choice of priors and Markov chain Monte Carlo settings
☒	☐	For hierarchical and complex designs, identification of the appropriate level for tests and full reporting of outcomes
☐	☒	Estimates of effect sizes (e.g. Cohen's *d*, Pearson's *r*), indicating how they were calculated

Our web collection on statistics for biologists contains articles on many of the points above.

Software and code

Policy information about availability of computer code

Data collection	For 16S rRNA, data collection was performed using the open source, publicly available code from MG-RAST (mg-download.py)<br>For genomes, they were manually downloaded using wget from the RefSeq repository.
Data analysis	All software used in this work is publicly available. All sources and versions of each software are stated in the manuscript.<br>For the table analysis, no specific code was produced. All analyses were performed using R.

For manuscripts utilizing custom algorithms or software that are central to the research but not yet described in published literature, software must be made available to editors/reviewers. We strongly encourage code deposition in a community repository (e.g. GitHub). See the Nature Research guidelines for submitting code & software for further information.

Data

Policy information about availability of data

All manuscripts must include a data availability statement. This statement should provide the following information, where applicable:

- Accession codes, unique identifiers, or web links for publicly available datasets
- A list of figures that have associated raw data
- A description of any restrictions on data availability

all data used in this work is publicly available. The sources and identifiers are all stated in the manuscript, in the form of supplementary data

Field-specific reporting

Please select the one below that is the best fit for your research. If you are not sure, read the appropriate sections before making your selection.

Ecological, evolutionary & environmental sciences study design

All studies must disclose on these points even when the disclosure is negative.

Study description	in this study we have analyzed the association between ecological features associated to extracellular proteins, and how they affect their evolution.
Research sample	We used all publicly available bacterial genomes from NCBI database (last accessed Nov. 2016) and all publicly available 16S rRNA datasets (last accessed march 2015)
Sampling strategy	no sampling strategy was performed. All data was collected from the same two repositories and treated equally.
Data collection	Data Collection was performed by both authors. Garcia-Garcera collected and curatedthe 16S rRNA information. Rocha collected and curated the genome data
Timing and spatial scale	not applicable, as this is a retrospective analysis. No specific sampling was performed.
Data exclusions	16S rRNA with less than 2000 sequences or with less than 85% of reads assigned to the 16S rRNA gene were excluded<br>Genomes belonging to phyla with less than 50 sequenced genomes, and genomes with poor phylogenetic classification were removed from the analysis
Reproducibility	Since all data is publicly available, this work is completely reproducible, when using the same datasets. With a greater number of datasets, we expect that a higher degree of resolution should be achieved, which might change the conclusions of this study
Randomization	not applicable
Blinding	not applicable

Did the study involve field work? ☐ Yes ☒ No

Reporting for specific materials, systems and methods

We require information from authors about some types of materials, experimental systems and methods used in many studies. Here, indicate whether each material, system or method listed is relevant to your study. If you are not sure if a list item applies to your research, read the appropriate section before selecting a response.

Materials & experimental systems

n/a	Involved in the study	
☒	☐	Antibodies
☒	☐	Eukaryotic cell lines
☒	☐	Palaeontology
☒	☐	Animals and other organisms
☒	☐	Human research participants
☒	☐	Clinical data

Methods

n/a	Involved in the study	
☒	☐	ChIP-seq
☒	☐	Flow cytometry
☒	☐	MRI-based neuroimaging
