# Supplementary material for: Community diversity and habitat structure shape the repertoire of extracellular proteins in bacteria
Source: Nat Commun. 2020 Feb 6;11:758. doi: 10.1038/s41467-020-14572-x (PMC7005277; doi:10.1038/s41467-020-14572-x)
Supplement: Supplementary file 4 — Description of Additional Supplementary Files [file 41467_2020_14572_MOESM4_ESM.pdf]

Description of Additional Supplementary Files

File Name: Supplementary Data 1
Description: List of metagenomic datasets included in the analysis, and their environmental classification.

File Name: Supplementary Data 2
Description: Values of alpha diversity associated to each meta-genomic dataset included in the analysis.

File Name: Supplementary Data 3
Description: Presence-Absence of bacterial species in the different environments.
The column "habitat specificity" is associated to species found only one environment. And the number represents the score of environmental structure associated to the environment where they were found.

File Name: Supplementary Data 4
Description: List of complete genomes included in the analysis.

File Name: Supplementary Data 5
Description: Average number of extracellular proteins per species.

File Name: Supplementary Data 6
Description: Average number of degradative enzymes and bacterio-cins associated to each bacterial species.

File Name: Supplementary Data 7
Description: HMM profiles for degradative enzymes and bacteriocins.
